# Prognostic utility and characteristics of MIB-1 labeling index as a proliferative activity marker in childhood low-grade glioma: a retrospective observational study

**DOI:** 10.1007/s00432-024-05701-w

**Published:** 2024-04-05

**Authors:** David Gorodezki, Julian Zipfel, Andrea Bevot, Thomas Nägele, Martin Ebinger, Martin U. Schuhmann, Jens Schittenhelm

**Affiliations:** 1 Department of Hematology and Oncology, University Children’s Hospital Tübingen, Tübingen, Germany; 2 Department of Neurosurgery, Section of Pediatric Neurosurgery, University Hospital Tübingen, Tübingen, Germany; 3 Department of Neuropediatrics and Developmental Neurology, University Hospital Tübingen, Tübingen, Germany; 4 Department of Neuroradiology, University Hospital Tübingen, Tübingen, Germany; 5 Institute of Pathology, Department of Neuropathology, University Hospital Tübingen, Tübingen, Germany

**Keywords:** Low-grade glioma, Proliferation index, MIB-1, KI-67

## Abstract

**Purpose:**

The prognostic utility of MIB-1 labeling index (LI) in pediatric low-grade glioma (PLGG) has not yet conclusively been described. We assess the correlation of MIB-1 LI and tumor growth velocity (TGV), aiming to contribute to the understanding of clinical implications and the predictive value of MIB-1 LI as an indicator of proliferative activity and progression-free survival (PFS) in PLGG.

**Methods:**

MIB-1 LI of a cohort of 172 nonependymal PLGGs were comprehensively characterized. Correlation to TGV, assessed by sequential MRI-based three-dimensional volumetry, and PFS was analyzed.

**Results:**

Mean MIB-1 LI accounted for 2.7% (range: < 1–10) and showed a significant decrease to 1.5% at secondary surgery (*p* = .0013). A significant difference of MIB-1 LI in different histopathological types and a correlation to tumor volume at diagnosis could be shown. Linear regression analysis showed a correlation between MIB-1 LI and preoperative TGV (R^2^ = .55, *p* < .0001), while correlation to TGV remarkably decreased after incomplete resection (R^2^ = .08, *p* = .013). Log-rank test showed no association of MIB-1 LI and 5-year PFS after incomplete (MIB-1 LI > 1 vs ≤ 1%: 48 vs 46%, *p* = .73) and gross-total resection (MIB-1 LI > 1 vs ≤ 1%: 89 vs 95%, *p* = .75).

**Conclusion:**

These data confirm a correlation of MIB-1 LI and radiologically detectable TGV in PLGG for the first time. Compared with preoperative TGV, a crucially decreasing correlation of MIB-1 LI and TGV after surgery may result in limited prognostic capability of MIB-1 LI in PLGG.

## Introduction

Pediatric low-grade gliomas (PLGG) comprise a heterogenous set of central nervous system (CNS) tumors of glial and mixed glioneural histology, representing the most common brain tumor types in childhood and adolescence, and are strongly associated with alterations of the RAS/MAPK pathway (Sievert and Fisher 2009; Rickert and Paulus 2001; Ryall et al. 2020b). These tumors are commonly characterized by a benign clinical course, mostly associated with favorable overall-survival rates and a low risk of malignant transformation or metastatic dissemination (Greuter et al. 2021; Sievert and Fisher 2009; Avinash et al. 2019; Chamdine et al. 2016; Krishnatry et al. 2016). However, depending on the tumor location, complete tumor resection can be crucially limited, as constantly high rates of incompletely resected PLGG have been reported over the past decades despite continuous advances in neurosurgical technology (Wisoff et al. 2011; Bandopadhayay et al. 2014; Stokland et al. 2010; Gnekow et al. 2012). Particularly in patients with tumors of limited resectability in deep-seated midline locations, recurrent progressions, treatment sequalae and unsatisfactory tumor control by subsequent adjuvant therapies frequently provoke significant morbidity (Krishnatry et al. 2016; Sadighi et al. 2018; Armstrong et al. 2009; van Iersel et al. 2020).

Ambivalent and barely predictable progression patterns of PLGG complicate treatment decisions and issue a challenge in postoperative care and adjuvant management after incomplete resection, as previous studies report of progression-free survival (PFS) rates of 45–65% after incomplete resection (Sievert and Fisher 2009; Wisoff et al. 2011; Stokland et al. 2010; Gnekow et al. 2012; Benesch et al. 2006; Jones et al. 2018; Ryall et al. 2020a; Fisher et al. 2001; Shaw and Wisoff 2003). Evaluation of reliable histological, molecular or imaging-based biomarkers for risk stratification is the objective of current research and ongoing clinical trials.

Molecular Immunology Borstel (MIB-1) is a monoclonal antibody used for qualitative detection of KI-67, a nonhistone DNA-binding nuclear protein exclusively expressed during active cell cycle phases, whereas showing absence during resting phase and senescence in paraffin-embedded sections (Gerdes et al. 1983; McCormick et al. 1993). Comparable to distinct CNS and solid non-CNS malignancies, the proportion of KI-67 expressing cells has repeatedly been described as a marker of the proliferative potential in glial tumors, while fostered by its unelaborate application by immunohistochemistry in formalin-fixed, paraffin-embedded (FFPE) tissues, nowadays plays an integral role in histopathological routine diagnostics (Schröder et al. 1991; Kałuza et al. 1997; Thotakura et al. 2014).

Although a correlation to histological grade in pediatric glioma has repeatedly been reported, several studies furthermore conformably show an association of KI-67/MIB-1 LI and PFS in pediatric high-grade glioma (Yao et al. 2023; Matsumoto et al. 1998; Pollack et al. 2002, 1997; Ho et al. 1998).

The prognostic significance of MIB-1 labeling index (LI) in PLGG, however, remains unclear, as previously published data of smaller case series partly show contradictory results and draw conflicting conclusions (Bowers et al. 2002, 2003; Fisher et al. 2002; Dorward et al. 2010; Margraf et al. 2011; Tu et al. 2018; Horbinski et al. 2010; Cherlow et al. 2019; Cler et al. 2022). In 2003, Bowers et al. reported of a study including 118 Pilocytic astrocytoma (PA) patients, showing a shortened PFS in patients with tumors bearing a MIB-1 LI > 2%. This data is in line with four subsequent studies on PAs including 35–80 cases, reporting a significant negative correlation of KI-67/MIB-1 LI and PFS (Fisher et al. 2002; Dorward et al. 2010; Margraf et al. 2011; Tu et al. 2018). In contrast, Horbinski et al. (2010) published a case series including 118 patients, showing no correlation of MIB-1 LI and adverse treatment outcome including recurrence, progression, metastatic spread or death. Similar results were reported by Cherlow et al. (2019) of 85 PLGG patients included in the ACNS0221 trial, as well as by Cler et al. (2022), showing no correlation of KI-67 with PFS in a series of PAs. Comparable results could be shown by Bowers et al. (2002) in a small series of pediatric low-grade oligodendrogliomas. It should be pointed out, that the previously published datasets almost exclusively are confined to distinct PLGG types, in most cases to PA, and follow-up data is often very limited. Despite its integral part in diagnostic routine, the overall predictive value of MIB-1 LI in PLGG remains unclear.

In this study, we aim to contribute data of a large representative single center PLGG cohort comprising various PLGG types. Beyond a characterization of MIB-1 LI values and analysis of a possible association with PFS and molecular data, we assess the correlation of MIB-1 LI values and pre- and postoperative tumor growth velocity, aiming to contribute to the understanding of the clinical implication and predictive value of MIB-1 LI as a surrogate marker of the proliferative activity in PLGG on progression-free survival.

Despite a growing understanding of distinct molecular features of subordinate tumor types, PLGG are, based on similar clinical characteristics, mainly managed by uniform diagnostic pathways and treatment algorithms (Gnekow et al. 2019). Therefore, an integrated analysis of the prognostic value of MIB-1 LI in PLGG comprising several tumor types may provide the highest comprehensive value for clinical practice. To take account of the distinct biology of various tumor types included in this cohort, however, we moreover compare tumor type-specific MIB-1 LI values and analyze the correlation of MIB-1 LI and PFS of various tumor types.

## Patients and methods

### Patient selection criteria

Patients < 18 years of age treated with histologically confirmed PLGG between 2006 and 2022 at University Children’s Hospital Tuebingen, a tertiary care referral center for pediatric neurosurgery and neuro-oncology, were identified by search of the medical center database and included to the study. Eligible diagnoses included glial and glioneural tumors CNS WHO grade 1 or 2 according to the 5th edition of the WHO classification of central nervous system tumors of 2021 (Louis et al. 2021) with exclusion of ependymomas. As feasible, pre-2021 tumor diagnoses were adopted to the currently valid classification based on available molecular genetic data by individually reviewing potentially relevant cases. Patients who did not undergo surgical treatment or received surgery at a foreign institution had to be excluded from subsequent analyses owing to unavailable histopathological data.

Histopathology reports were contributed by the Department of Neuropathology at University Hospital Tuebingen, while diagnoses were routinely confirmed by the German Brain Tumor Reference Center (Institute of Neuropathology, University of Bonn Medical Center, Bonn, Germany). Patients diagnosed with PLGG associated with Neurofibromatosis Type 1 (NF-1) and other phacomatoses were equally included into the study.

### Methods

Demographical and clinical data of eligible patients including age, sex, histopathological diagnosis, molecular BRAF status, tumor site and chronological sequence of events were extracted from the Pediatric Neuro-oncology center database.

Analysis of pre- and postoperative tumor growth velocity was implemented using serial quantification of tumor burden on sequentially acquired T2-weighted MRI scans, in most cases by 1.5/3 T MRI scanners. Due to previous experience in distinct intracranial malignancies, three-dimensional volumetry was preferred to linear assessment of lesion extension, as comparative analysis has shown superior sensitivity of three-dimensional assessment of tumor expansion (Harris et al. 2008). Therefore, semiautomated calculation of tumor volume was conducted following manual determination of tumor margins in 1–3 mm axial MRI slices. Image based volumetric segmentation was applied using BrainLab Elements (version 3.0, BrainLab, Munich, Germany), a specialized and broadly applied software for image guided therapy in surgical and radiation oncology. Tumor growth velocity was quantified by calculation of tumor expansion over time. Repeated volumetry of various investigators showed negligible variation of tumor volumes.

MIB-1 LI was routinely assessed at the time of histopathological diagnosis following automated immunohistochemical staining of slides from paraffin-embedded tumor samples using a well-established KI-67 antibody (DakoCytomation, Glostrup, Denmark, clone MIB-1, dilution 1:200). Therefore, an automated staining application (BenchMark®, Ventana Medical Systems, Tucson, Az, USA) was used, applying cell condition pretreatment (CC1 for 40 min, antibody incubation at 42 °C for 20 min) and using a universal biotinylated immunoglobulin secondary antibody, combined with diaminobenzidine as a substrate. Nuclear counterstaining with hemalaun was applied. Evaluation was implemented by various experienced neuropathologists at the time of initial diagnosis. For the purpose of a maximally achievable homogeneity, MIB-1 LI assessment was carried out by calculating the overall percentage of MIB-1 positive tumor nuclei on large-scale full slide sections including proliferation hotspots without confining to areas of high proliferative activity. Fainted colored nuclei were categorically counted as positive. In case of significant disparities of estimated MIB-1 LI values, individual discussion and reassessment was conducted. MIB-1 LI values were later retrieved from pathological records during data acquisition for the present study.

Testing for BRAF V600E mutation was performed at time of diagnosis via pyrosequencing following PCR amplification of the BRAF gene from extracted tumor DNA. Testing for BRAF-KIAA1549 fusion was performed at time of diagnosis after extraction of RNA from tumor material and subsequent reverse transcription into cDNA by use of fusion transcript specific primers and electrophoretic segregation.

### Statistical analyses

Statistical analysis of the reported data was conducted using JMP 15.2.0 (SAS Institute Inc., Cary, North Carolina, USA) and GraphPad Prism 8.0 (GraphPad Software, Inc., California, USA). Anderson − Darling test was used to study distribution of MIB-1 LI values and pre- and postoperative tumor growth rates. Owing to not normally distributed data, nonparametric testing was conducted for further statistical analysis using Mann − Whitney rank sum test and Kruskal − Wallis test. Log-rank (Mantel-Cox) test was performed for PFS curve comparison. *P* values < 0.05 were considered statistically significant.

## Results

During the observation period, 191 patients were treated with PLGG at the stated institution. Patient age ranged from 2 to 17 years (mean: 7.9 years). Diagnoses included Pilocytic astrocytoma °1 (139 cases), Ganglioglioma °1 (36 cases), Pediatric-type diffuse low-grade glioma °2 (14 cases), Oligodendroglioma °2, IDH-mutant, 1p/19q codeleted (2 cases), Pleomorphic xanthoastrocytoma °2 (1 case), Rosette-forming glioneural tumor °1 (1 case) and Subependymal giant cell astrocytoma °1 (1 case). As several cases including diffuse low-grade glioma °2 were diagnosed up to seventeen years ago, a precise re-classification in accordance with the latest edition of the WHO classification appeared unfeasible in several cases. Among these tumors, either IDH1/2 mutations and MAPK alterations were found in three cases, respectively. Reviewing the available molecular data, these cases presumably include MAPK altered pediatric-type diffuse low-grade gliomas °2 and MAPK altered and MYB-/MYBL-altered pediatric-type diffuse astrocytoma °2. Detailed results of the molecular BRAF analyses of this cohort have previously been published in a comprehensive analysis of the tumor growth velocity of the reported cohort (Gorodezki et al. 2022). Association to NF-1 was present in 23 cases. Tumor locations included the posterior fossa (PF, 80 cases), the supratentorial midline and optic nerve (SML and OG, 55 cases), the cerebral hemispheres (CH, 46 cases), the spinal cord (SC, 8 cases) and the lateral ventricles (LV, 2 cases). Of 172 patients (90.1%) receiving surgery during the observation period, gross-total resection (GTR) could be achieved in 65 cases (37.7%), while incomplete resection (IR) was in realized in 100 cases (58.1%). Biopsy was carried out in 7 cases (4.1%). A total of 38 patients (22.1%) received repeated surgery during the observation period, while adjuvant treatment including chemotherapy, radiation or targeted therapy was applied in 23 patients (12%). Because our institution serves as a referral center for pediatric neurosurgery, in 18 cases (10.5%) first surgery was performed at a foreign institution, hence detailed histopathological records including MIB-1 LI values on the first tumor were not available for analysis. Distribution of diagnoses, tumor sites and treatment patterns showed congruency to previously published population based PLGG cohorts (Bandopadhayay et al. 2014; Stokland et al. 2010; Gnekow et al. 2012).

### MIB-1 labeling index: distribution, characterization and treatment-dependent sequence

**Fig. 1:**
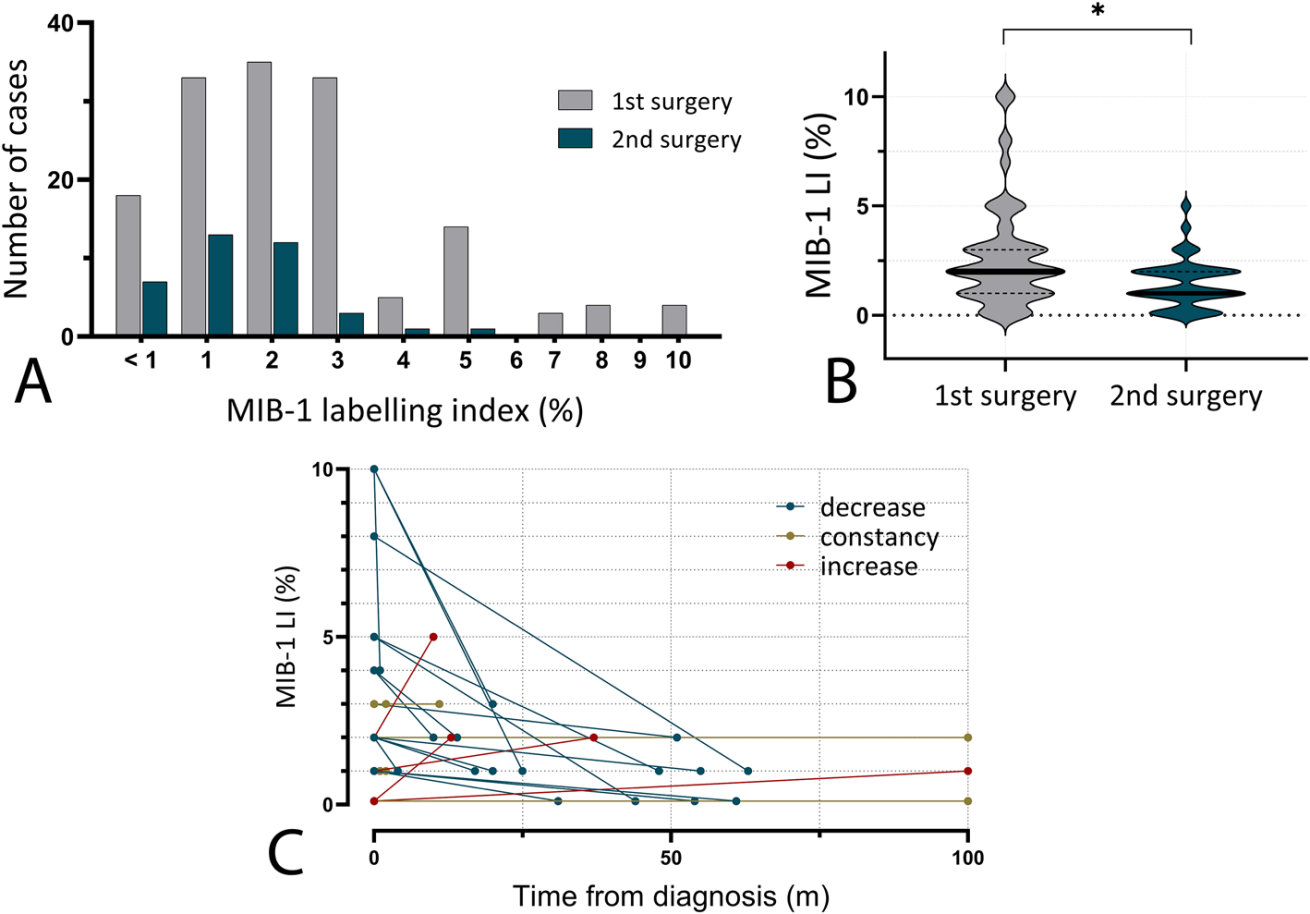
**A** Distribution of MIB-1 LI values at 1st and 2nd surgery of a large single center PLGG cohort **B** Comparison of mean MIB-1 values at 1st and 2nd surgery showed a significant difference (2.7 vs 1.5%, respectively, Mann − Whitney *U* test, *p* = 0.0013) **C** Individual chronological sequences of MIB-1 LI values of 27 patients receiving two consecutive surgeries. In 19 patients (70.4%), a decrease of individual MIB-1 LI values could be observed, while an increase of MIB-1 LI could only be detected in 4 cases (14.8%)

Distribution of MIB-1 LI values at first and second surgery are illustrated Fig. 1A. MIB-1 LI values showed a mean of 2.7% at first surgery (range: < 1–10%, *n* = 154), while mean MIB-1 LI at second surgery accounted for 1.5% (< 1–5%, *n* = 38). Comparison of median values showed a significant difference (Mann − Whitney *U* test, *p* = 0.0013, see Fig. 1B). In 27 patients who underwent repeated surgeries, available MIB-1 LI values at the time of 1st and 2nd surgery allowed for individual chronological outline of MIB-1 LI during individual treatment periods. In 19 patients (70.4%), a decrease of individual MIB-1 LI values could be observed, while an increase of MIB-1 LI could only be detected in 4 cases (14.8%). Individual chronological sequences of MIB-1 LI values are illustrated in Fig. 1C.

**Fig. 2:**
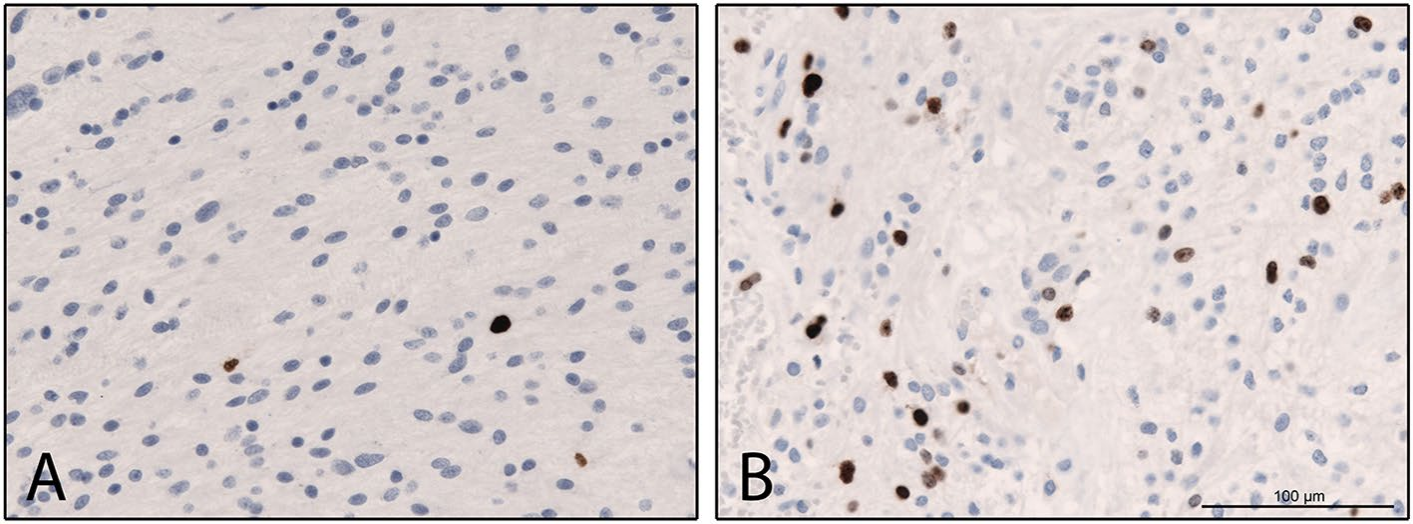
Comparative illustration of two cases of Pilocytic astrocytoma °1 showing a varying fraction of KI-67 expressing nuclei after immunohistochemical MIB-1 staining (**A**: MIB-1 LI = 1%; **B**: MIB-1 LI = 3%; Ki67, clone MIB1, Dako Glostrup, 1:200 magnification, Ventana immunohistochemistry system, diaminobenzidine as brown chromogen)

**Fig. 3:**
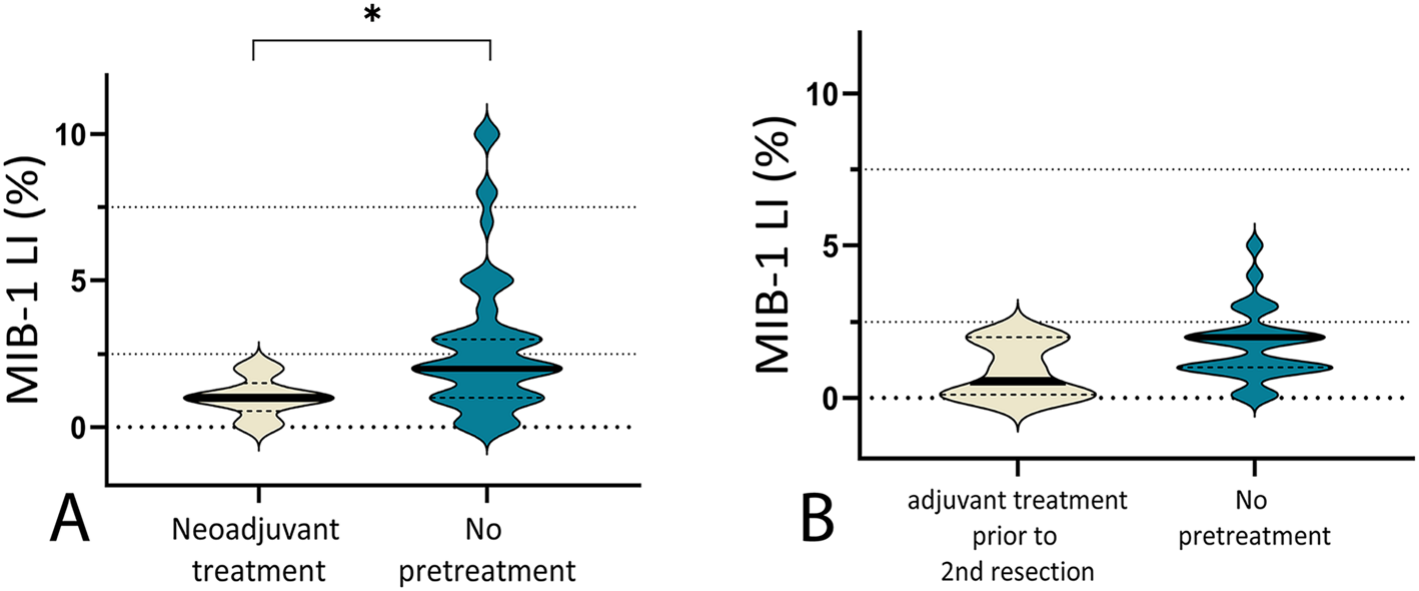
**A** Comparative analysis of MIB-1 LI values of pretreated vs treatment naïve PLGG at 1st surgery showed significantly lower mean MIB-1 LI values in patients pretreated with neoadjuvant radio-/chemotherapy (1.0 vs 2.8%, Mann − Whitney *U* test, *p* = 0.035) **B** At 2nd surgery, comparison of MIB-1 LI values of pretreated vs treatment naïve tumors showed no statistically significant difference (0.9% vs 1.7%, Mann − Whitney *U* test, *p* = 0.11, see Fig. 2B)

We furthermore compared mean MIB-1 LI values of pretreated vs treatment-naïve PLGG, aiming to characterize the impact of neoadjuvant treatment on MIB-1 LI. Prior to first surgical intervention, a total of five patients received neoadjuvant radio-/chemotherapy, while no pretreatment was applied in 140 patients. Compared with treatment-naïve PLGG, pretreated tumors showed a significantly lower mean MIB-1 LI value (1.0 vs 2.8%, Mann − Whitney *U* test, *p* = 0.035, see Fig. 3A). At 2nd surgery, comparison of MIB-1 LI values of pretreated vs treatment naïve tumors showed no statistically significant difference (0.9% vs 1.7%, Mann − Whitney *U* test, *p* = 0.11, see Fig. 3B).

Studying age dependence of MIB-1 LI revealed significantly higher MIB-LI values in younger patients (3.1 vs 2.8 vs 2.4% in patients aged 0–5 vs 6–11 vs 12–18 years at time of diagnosis, respectively,* p* = 0.04, Kruskal − Wallis test). Comparison of tumor type-specific MIB-1 LI values showed a significant difference, as Pilocytic astrocytomas °1 were characterized by the highest mean MIB-1 LI values, followed by pediatric-type diffuse low-grade gliomas (including diffuse astrocytomas °2, *MYB* or *MYBL1*-altered; and diffuse low-grade gliomas °2, *MAPK*-pathway altered) and Gangliogliomas °1 (2.9 vs 2.6 vs 2.1%, *p* = 0.04, Kruskal − Wallis test). Remarkably, tumors characterized by an initial volume of > 20 cm^3^ at diagnosis showed a significantly higher mean MIB-1 LI as compared to tumors showing a volume of ≤ 20 cm^3^ (3.6 vs 2%, *p* = 0.002, Mann − Whitney *U* test).

No significant differences of mean MIB-1 LI values could be shown regarding to patient sex, various tumor locations, WHO grade and the most frequent molecular aberrations of BRAF in PLGG (BRAF-KIAA1549 fusion and BRAF V600E-mutation) compared to BRAF wild-type tumors. Patient characteristics and corresponding MIB-1 LI values are illustrated in Table 1.

**Table 1:** Patient characteristics and correlation between mean MIB-1 labelling index values and clinical, histological and molecular variables

	MIB-1 labelling index (%)		
Variable	*n* *(%)*	Mean (SD)	*P* value
*Age*			0.04
0–5	40 (27.6)	3.1 (2.3)	
6–11	43 (29.6)	2.8 (2.3)	
12–18	62 (42.8)	2.4 (2.5)	
*Sex*			0.49
Female	67 (46.2)	2.8 (2.6)	
Male	78 (53.8)	2.7 (2.1)	
*Tumor site*			0.32
Posterior fossa	66 (45.5)	2.7 (2.4)	
Cerebral hemispheres	45 (31)	2.2 (2.1)	
SMG and OPG^a^	30 (20.7)	2.7 (2.4)	
Spinal cord	5 (3.5)	1.8 (1.3)	
Lateral ventricle	2 (1.4)	4.5 (3.5)	
*Tumor size at diagnosis*			0.002
≤ 20 cm^3^	68 (46.9)	2 (1.5)	
> 20 cm^3^	77 (53.1)	3.6 (2.8)	
*Diagnosis*			0.04
Pilocytic astrocytoma °1	96 (66)	2.9 (2.2)	
Ganglioglioma °1	31 (21.4)	2.1 (2.4)	
Pediatric-type diffuse low-grade glioma °2^b^	14 (9.7)	2.6 (2.7)	
Other^c^	5 (2.8)	2.8 (1.5)	
*WHO grade*			0.18
Grade 1	129 (89)	2.7 (2.4)	
Grade 2	16 (11)	2.5 (2.5)	
*BRAF V600E*			0.38
Wild-type	71 (79.8)	2.6 (2.5)	
Mutant	18 (20.2)	2.8 (2.4)	
*BRAF-KIAA1549 fusion*			0.26
Positive	31 (34.8)	2.9 (2.3)	
Negative	58 (65.2)	2.5 (2.6)	

^a^Supratentorial midline glioma and optic pathway glioma

^b^Including: Diffuse astrocytoma °2, *MYB* or *MYBL1*-altered; and diffuse low-grade glioma °2, *MAPK*-pathway altered

^c^Including: Pleomorphic xanthoastrocytoma °2 (1), Rosette-forming glioneural tumor °1 (1), Supependymal giant cell astrocytoma °1 (1), Oligodendroglioma °2, IDH-mutant, 1p/19q codeleted (2)

### Correlation of MIB-1 labeling index on pre- and postoperative tumor growth velocity

To study the clinical significance of MIB-1 LI as a proliferative activity marker in PLGG, we analyzed the implication of MIB-1 LI on pre- and postoperative tumor growth velocity within our cohort.

**Fig. 4:**
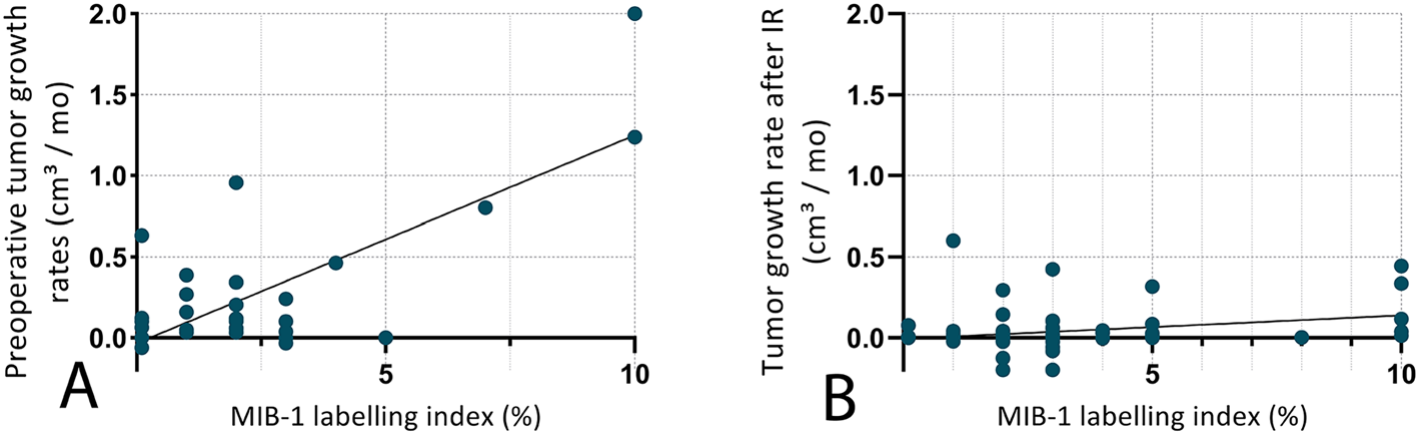
**A** Linear regression analysis showed a significant correlation between MIB-1 LI values and preoperative tumor growth velocity (*n* = 31, *R* = 0.128, *R*^2^ = 0.546, *p* < 0.001) **B** A significantly decreasing correlation of MIB-LI and postoperative tumor growth velocity could be shown (*n* = 76, *R* = 0.014, *R*^2^ = 0.08, *p* = 0.013)

In 31 patients, comparable MRI sequences over a surveillance period of ≥ 6 months prior to surgical resection or biopsy allowed for calculation of preoperative tumor growth rates. Linear regression analysis showed a significant correlation between MIB-1 LI values and preoperative tumor growth velocity (*n* = 31, *R* = 0.128, *R*^2^ = 0.546, *p* < 0.001, see Fig. 4A).

For calculation of postoperative tumor growth rates, comparable sequential postoperative MRI data of a surveillance period of ≥ 6 months with corresponding MIB-1 LI values of a total of 76 patients could be included to the subsequent analyses. Compared to preoperative tumor growth rates, a crucially decreasing correlation of MIB-1 LI values and postoperative tumor growth velocity could be shown after IR (linear regression analysis, *n* = 76, *R* = 0.014, *R*^2^ = 0.08, *p* = 0.013, see Fig. 4B).

### Correlation of MIB-1 labeling index and progression-free survival (PFS)

We furthermore studied the correlation of MIB-1 LI values and PFS within the reported cohort after IR and GTR. Including all patients, 5- and 10-year PFS after IR accounted for 63% and 46%, respectively, while 5- and 10-year PFS after GTR accounted for 91%.

**Fig. 5:**
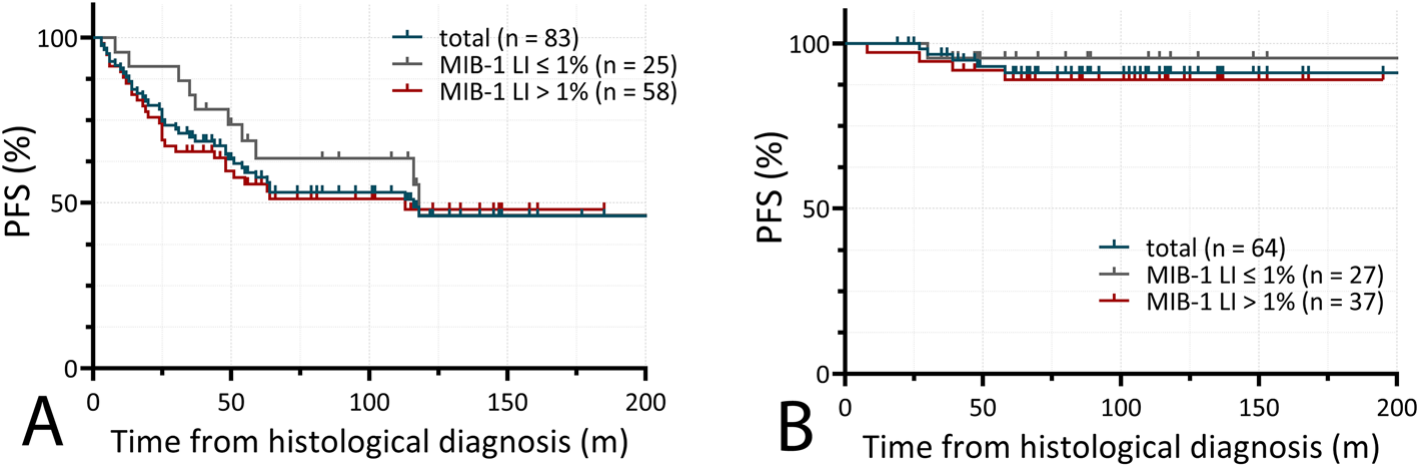
**A** Comparison of 5- and 10-year-PFS in incompletely resected PLGG with MIB-1 LI ≤ 1 vs > 1% showed no significant difference (63.5 vs 55.6% and 46.2 vs 47.1%, respectively, log rank test, Chi square 0.63, *p* = 0.625) **B** No significant difference of 5- and 10-year-progression-free survival in gross-totally resected PLGG with MIB-1 LI ≤ 1 vs > 1% could be shown (95.5 vs 89.0%, respectively, log rank test, Chi square 0.58, *p* = 0.75)

Within the subgroup of patients undergone IR, comparison of 5- and 10-year PFS in cases with MIB-1 LI ≤ 1 vs > 1% showed no significant disparity, as 5-year PFS accounted for 63.5 vs 55.6%, respectively, while 10-year PFS accounted for 46.2 vs 47.1% (*n* = 83, log rank test, Chi square = 0.63, *p* = 0.625, see Fig. 5A).

After GTR, equally no significant difference in 5- and 10-year PFS in patients with MIB-1 LI ≤ 1 vs > 1% could be observed, as 5- and 10-year PFS accounted for 95.5 vs 89.0%, respectively (*n* = 64, log rank test, Chi square = 0.58, *p* = 0.75, see Fig. 5B).

**Fig. 6:**
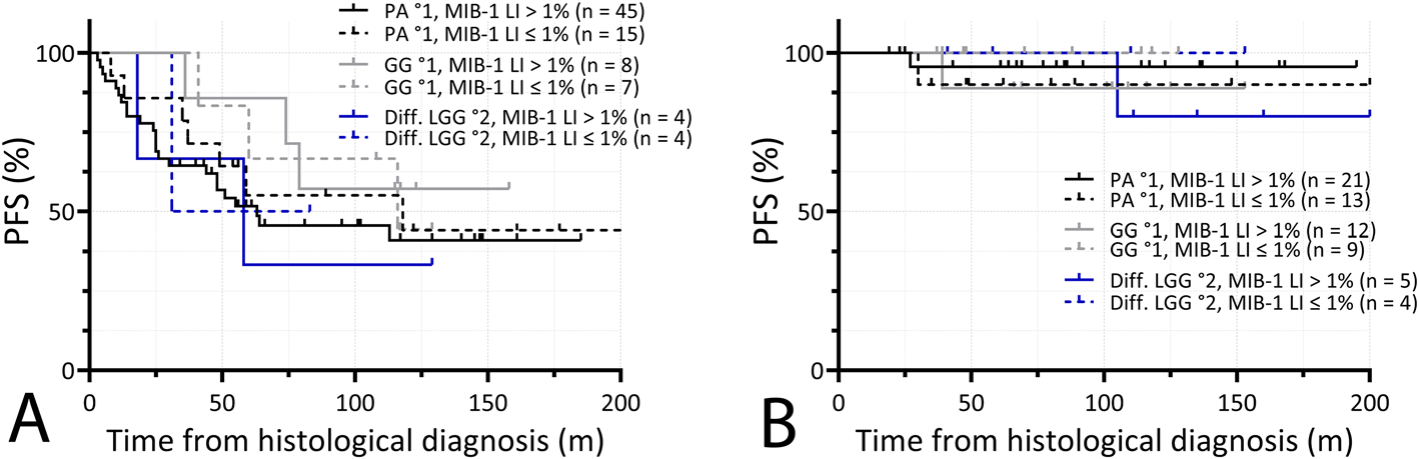
**A** Tumor type specific comparison of PFS of tumors bearing a MIB-1 LI > 1 vs ≤ 1% after incomplete resection showed no significant difference in Pilocytic astrocytomas °1, pediatric-type diffuse low-grade gliomas or ganglioglioma °1. **B** In patients who received gross-total resection, tumor type specific comparison of PFS of tumors bearing a MIB-1 LI > 1 vs ≤ 1% after gross-total resection likewise showed no significant difference in Pilocytic astrocytomas °1, pediatric-type diffuse low-grade gliomas or ganglioglioma °1

Tumor type specific comparison of PFS of tumors bearing a MIB-1 LI > 1 vs ≤ 1% after incomplete resection showed no significant difference in Pilocytic astrocytomas °1 (5-year PFS 45 vs 45%, respectively, log-rank test, Chi square = 0.32, *p* = 0.57, *n* = 60), pediatric-type diffuse low-grade gliomas (5-year PFS 33 vs 50%, respectively, log-rank test, Chi square = 0.08, *p* = 0.78, n = 8) or ganglioglioma °1 (5-year PFS 85 vs 66%, respectively, log-rank test, Chi square = 0.04, *p* = 0.84, *n* = 15). Respective Kaplan − Meier curves are illustrated in Fig. 6A.

Comparison of PFS of tumors bearing a MIB-1 LI > 1 vs ≤ 1% after gross-total resection likewise showed no significant difference in Pilocytic astrocytomas °1 (5-year PFS 96 vs 90%, respectively, log-rank test, Chi square = 0.35, *p* = 0.56, *n* = 34), pediatric-type diffuse low-grade gliomas (5-year PFS 100%, respectively, log-rank test, Chi square = 0.40, *p* = 0.53, *n* = 9) or ganglioglioma °1 (5-year PFS 89 vs 100%, respectively, log-rank test, Chi square = 0.89, *p* = 0.35, *n* = 21). Respective Kaplan–Meier curves are illustrated in Fig. 6B.

## Discussion

In the present work, beyond studying its prognostic utility on a large representative single-center PLGG cohort, we aim to contribute to a more nuanced understanding of the clinical implications of MIB-1 LI as a potential surrogate marker for the proliferative activity of PLGG. For this purpose, a potential association of MIB-1 LI values and pre- and postoperative tumor growth behavior has been analyzed including all potential cofounding factors. Quantification of tumor growth velocity has been conducted using sequential three-dimensional MRI based tumor volumetry, as this method has shown a superior sensitivity in growth tracking as compared to linear diameter measurements in intracranial tumors (Harris et al. 2008).

While nowadays amending an integral part to histopathological routine diagnostics in CNS tumors, assessment of MIB-1 LI has previously shown to contribute to differentiation of the degree of malignancy in tumors of the central nervous system, while a significant correlation to WHO grade in human glioma has been reported (Matsumoto et al. 1998; Pollack et al. 2002; Skjulsvik et al. 2014; Hsu et al. 1997; Krishnan et al. 2019). However, as compared to distinct solid CNS and non-CNS malignancies, previous analyses of smaller PLGG cohorts addressing the prognostic value of KI-67/MIB-1 LI in PLGG, many of them from the pre-molecular era, draw conflicting conclusions and necessitate further assessment (Bowers et al. 2002, 2003; Fisher et al. 2002; Dorward et al. 2010; Margraf et al. 2011; Tu et al. 2018; Horbinski et al. 2010; Cherlow et al. 2019; Cler et al. 2022).

Analysis of a potential coherence between the fraction of MIB-1 positive cells and the growth velocity within the analyzed cohort of PLGG notably showed a significant correlation of MIB-1 LI and both pre- and postoperative radiologically assessed tumor growth rates, as illustrated in Fig. 4. This observation may possibly confirm a clinical significance of KI-67/MIB-1 LI as a surrogate marker for the proliferative activity of glioma cells, as originally shown in comprehensive analyses on a cellular level, leading to the establishment of MIB-1 LI as the commonly used method for measuring the proliferative potential in human gliomas (Schröder et al. 1991; Kałuza et al. 1997; Thotakura et al. 2014). In the current work, a significant correlation of MIB-1 LI and radiologically quantifiable tumor growth velocity could be shown for the first time.

A clinically significant correlation of MIB-1 LI and tumor growth may also be confirmed by the observation of a significant coherence of MIB-1 LI and tumor volume at diagnosis within the analyzed cohort, as gliomas showing a volume of > 20 cm^3^ at time of detection bearing a higher mean fraction of MIB-1 positive tumor cells compared to tumors measuring ≤ 20 cm^3^ at time of diagnosis, possibly indicating faster preoperative tumor growth velocity of PLGG bearing a higher MIB-1 LI.

Remarkably, however, compared to preoperative tumor growth rates, a crucially decreasing correlation of MIB-1 LI values at time of incomplete resection on postoperative tumor growth velocity could be shown, as illustrated in Fig. 4. This may be explained by a significant alteration of tumor growth velocity caused by surgical intervention, as recently published data indicates a significant deceleration of tumor growth in PLGG after surgical intervention, predominantly determined by the extent of resection (Gorodezki et al. 2022). Presumably, this significant alteration of tumor growth behavior by surgical intervention may be the cause for a limited predictive value of MIB-1 LI at time of incomplete resection regarding postoperative growth velocity, and subsequently resulting in a limited prognostic utility of MIB-1 LI regarding progression-free survival in PLGG. Eventually, no significant association of MIB-1 LI and long-term PFS could be shown both after incomplete and gross-total resection within the analyzed cohort. For this reason, we do not advocate the use of a MIB-1 LI cutoff outside of the neurosurgical context for tumor risk stratification.

In context of the previously published observation of growth deceleration after surgical intervention in this cohort, the significantly lower mean MIB-1 LI at time of secondary surgery shown in this analysis may possibly be seen as a coherence of growth deceleration and decrease of mean MIB-1 LI values (Gorodezki et al. 2022).

Characterization of MIB-1 LI in PLGG furthermore showed age dependence of mean MIB-1 LI values, as patients 0 – 5 years of age showed the highest mean MIB-1 LI value, with a decreasing tendency in sub-teenage and adolescent patients. An age dependence with a tendency towards higher MIB-1 LI values in PLGG particularly in infants has previously described, and may possibly be seen as an expression of young age representing a risk factor for significantly higher progression rates and worse treatment outcomes in PLGG (Bandopadhayay et al. 2014; Stokland et al. 2010; Gnekow et al. 2012; Fisher et al. 2002; Tu et al. 2018). The association of MIB-1 LI with age may also explain contradictory results in previous studies as the age composition of the respective cohorts may differ significantly.

Comparing mean MIB-1 LI values of distinct histologic tumor types showed a minor, thus significant difference, as PAs °1 showed the highest mean MIB-1 LI, while the lowest mean value was detected Ganglioglioma °1. In context of PA not showing a significantly unfavorable long-term PFS in recent population-based cohort studies, the described differentiating mean MIB-1 LI values of distinct histological diagnoses should presumably not be seen as prognostically relevant (Krishnatry et al. 2016; Wisoff et al. 2011; Bandopadhayay et al. 2014; Stokland et al. 2010; Gnekow et al. 2012).

Further analysis showed no significant association of patient sex, tumor location, WHO grade and detection the most frequent molecular aberrations of BRAF in PLGG (BRAF-KIAA1549 fusion and BRAF V600E-mutation) with MIB-1 LI values. A previously mentioned smaller case series of 70 PAs consistently showed no significant difference of mean MIB-1 LI values in tumors of various locations (Tu et al. 2018).

There are, however, limitations to this study to be addressed. First, it should be pointed out, that nonautomated assessment of MIB-1 LI values was applied, potentially bearing interobserver variability and limited accuracy of the analyzed data. Although a standardized protocol for immunohistochemical staining of MIB-1 and counting of LI has been applied, and estimation of MIB-1 LI furthermore has been carried out by various experienced neuropathologists, interobserver variability of MIB-1 LI values within the presented data should, to some degree, be presumed. Previous analyses pointed out significant interobserver variability of MIB-1 LI assessment in primary brain tumors, depending on applied counting methods and height of LI values, subsequently leading to the development of automated counting systems (Hsu et al. 2003; Grzybicki et al. 2001). Nevertheless, with nonautomated measurement of MIB-1 LI representing the most prevalently used method to this day, application of manual MIB-1 LI assessment may contribute to the transferability and viability of the analyzed data in neuropathology practice. Potentially limited accuracy and interobserver variability, moreover, should be considered a possible explanation of the partially conflicting results of previous studies on the prognostic utility of MIB-1 LI in PLGG (Bowers et al. 2002, 2003; Fisher et al. 2002; Dorward et al. 2010; Margraf et al. 2011; Tu et al. 2018; Horbinski et al. 2010; Cherlow et al. 2019; Cler et al. 2022).

Further limitations include the retrospective nature of the study, as a significant number of patients had to be excluded from the analyses due to limited availability of comparable MRI sequences for quantification of tumor growth within the follow-up period. However, that the importance of a solid reproducibility of a potentially subjective variable like the MIB-1 LI, which may be significantly influenced by inter-laboratory deviations, does support the application of a single center approach in the current study.

Although the single center approach of the study should be considered as another limitation, the distribution of diagnoses, tumor sites and treatment patterns showed congruency to previously published population based PLGG studies, underlining the representativity of the reported cohort (Bandopadhayay et al. 2014; Stokland et al. 2010; Gnekow et al. 2012).

## Conclusion

This data possibly confirms a significant correlation of MIB-1 LI and radiologically detectable tumor growth velocity in PLGG for the first time. However, compared to preoperative tumor growth rates, a crucially decreasing correlation of MIB-1 LI values and tumor growth rates after surgical intervention and age-dependent correlation could be shown, subsequently resulting in a limited prognostic value of MIB-1 LI cutoffs regarding PFS in PLGG.

## Data Availability

The datasets generated and analyzed during the current study are available from the corresponding author upon reasonable request.
